# Dot in circle sign on MRI in foot mycetoma

**DOI:** 10.1016/j.radcr.2024.02.004

**Published:** 2024-02-27

**Authors:** Yash Kashikar, Shivali Kashikar, Bhushan Madke, Soham Meghe, Kaveri Rusia

**Affiliations:** a Department of Dermatology, Venereology and Leprosy, Datta Meghe Institute of Higher Education and Research, Jawaharlal Nehru Medical College, Wardha, Maharashtra, India; b Department of Radiodiagnosis, Datta Meghe Institute of Higher Education and Research, Jawaharlal Nehru Medical College, Wardha, Maharashtra, India

**Keywords:** Mycetoma, Actinomycetoma, Foot, Imaging, Magnetic resonance

## Abstract

Mycetoma or Maduramycosis is a chronic granulomatous infectious condition encountered mostly in tropical and subtropical regions. It affects the deep subcutaneous tissues, which may progress to involve the muscles and bones later in the course of the disease. It can be caused by fungi (eumycetoma), and bacteria (actinomycetoma) predominantly affecting the foot. Demonstration of the causative agent by biopsy and microbiological studies helps to establish a confirmative diagnosis, and choosing correct antimicrobial therapy. However, it may be delayed resulting in increased patient morbidity. Thus, imaging plays a vital role in early recognition & prompt treatment, especially MRI which is a non-invasive procedure demonstrating the hallmark dot in circle sign. Here we report a case of mycetoma foot with pathognomic MRI findings.

## Introduction

A chronic (indolent) infection that mostly affects the deep subcutaneous tissues is called mycetoma, or Madura foot. The condition mainly affects poorer populations in remote rural areas in tropical and subtropical countries at altitudes between 30° North and 15° South, the so-called “mycetoma belt” regions, including Sudan, Somalia, Senegal, Yemen, India, Mexico, and Venezuela [1,2]. Clinically, it manifests as a triad of swelling, discharging sinuses, and the extrusion of distinctive grains (colonies of causative organisms). However, it usually takes months or several years to develop complete clinical signs and symptoms, which can cause a major delay in the diagnosis and course of treatment. In late complicated cases, amputation can be considered to stop the infection from spreading. Most of the patients have not received enough attention, due to their living in remote rural areas that lack enough medical services, including well-trained medical personnel, diagnostic facilities, and treatment options. Mycetoma is, therefore, recognized as a “neglected tropical disease” by the World Health Organization (WHO) [3]. Mapping the extent of disease and enabling successful therapy requires early diagnosis, and it could be possible through imaging studies.

## Case presentation

**Fig. 1:**
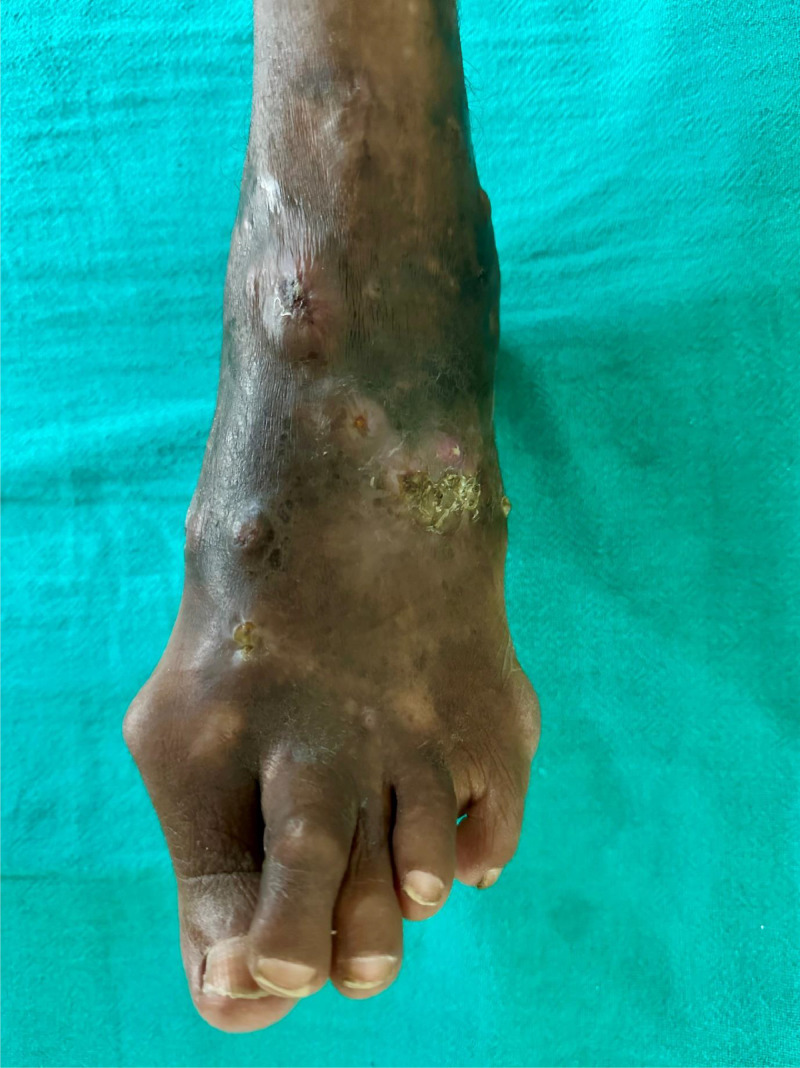
Diffuse indurated swelling of left foot having multiple sinuses with sero-purulent discharge over the dorsum of left foot. Hallus Valgus at the base of left great toe with overriding toes.

A 42-year-old female from central India presented at the Department of Dermatology of a tertiary care hospital with a 15-year history of painless swelling studded with multiple sinuses over her left foot. The swelling appeared after sustaining an injury by a wooden splinter while working outdoors in the fields. The patient was treated with oral antibiotics on multiple occasions in a nearby primary health center, with no relief from the symptoms and persistence of swelling. She did not have any co-morbidities and was not immunocompromised. Clinical examination revealed diffusely indurated swelling of the left foot having multiple sinuses with sero-purulent discharge over the dorsum and sole of the left foot along with bony deformities (Fig. 1).

**Fig. 2:**
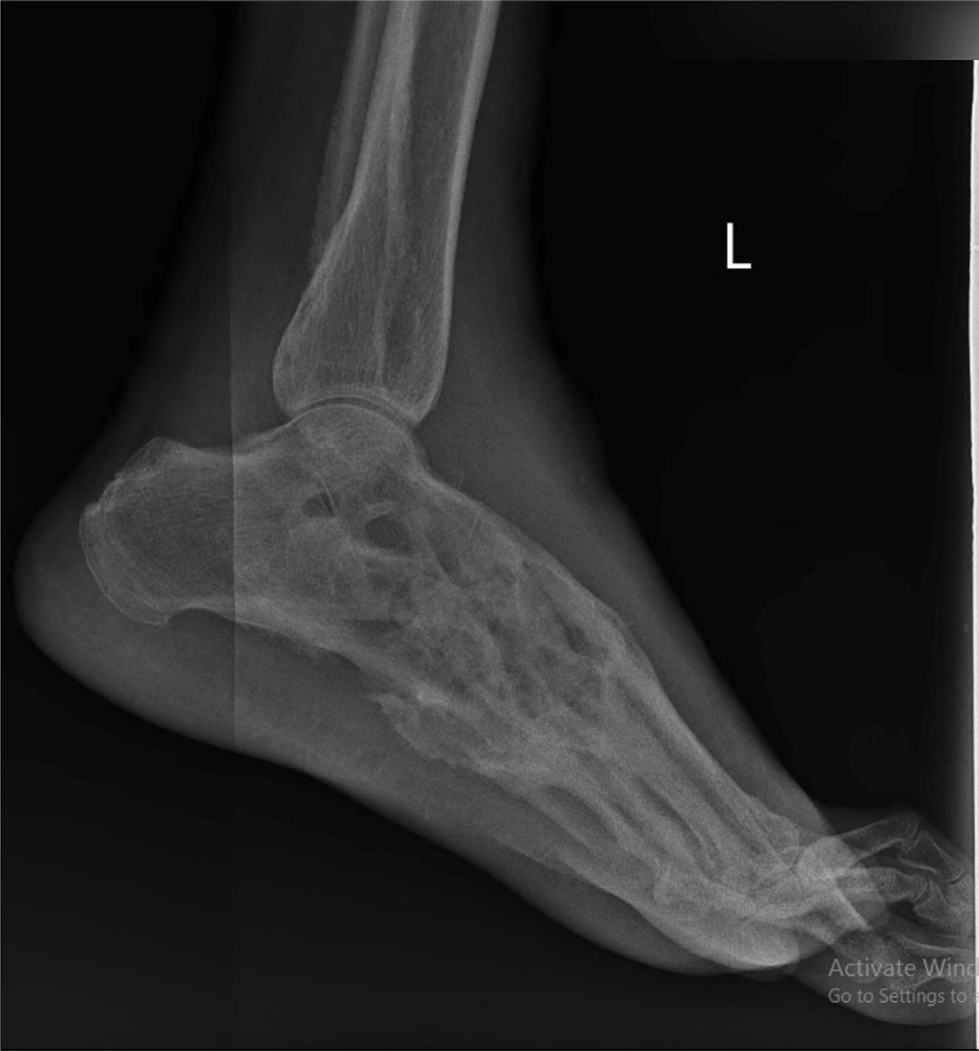
Radiograph foot (Lateral projection) showing ill-defined lytic and sclerotic lesions in tarsal bones with ankylosis at inter-tarsal and metatarsophalangeal joints.

**Fig. 3:**
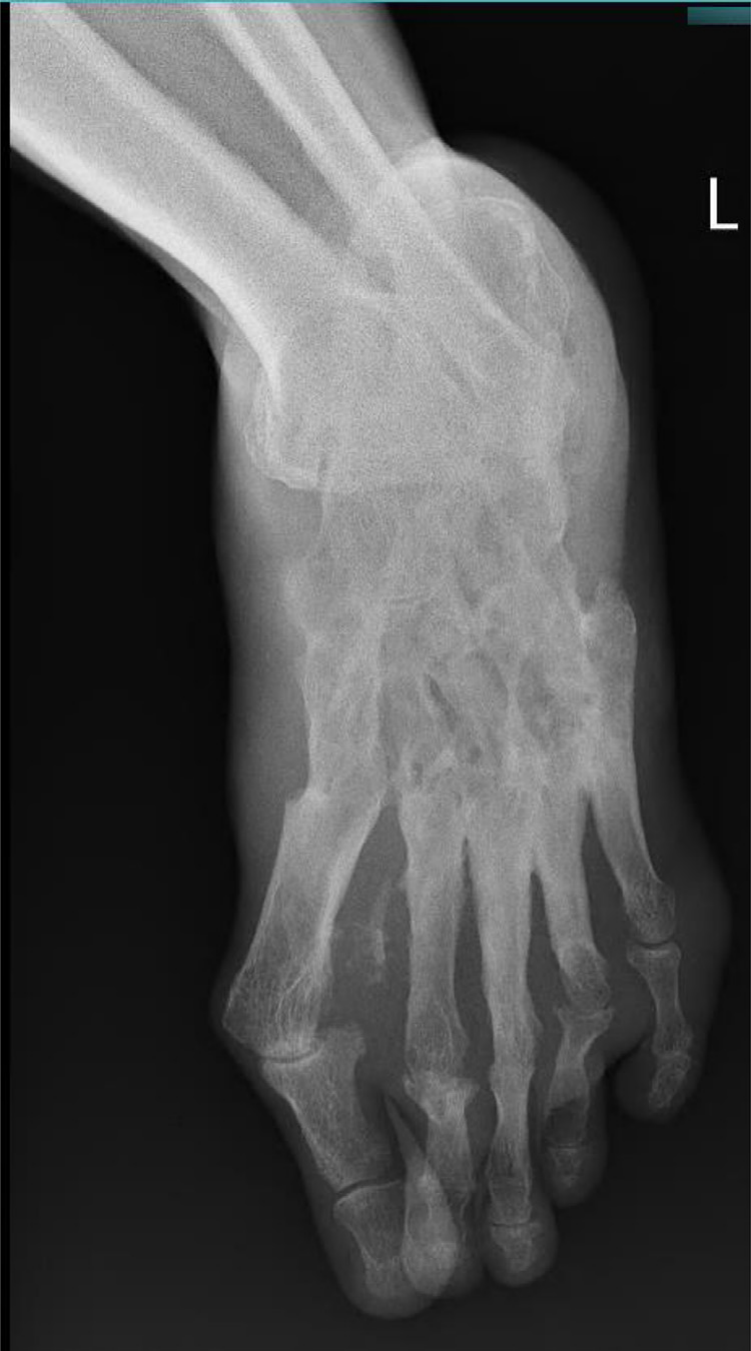
Radiograph foot (AP projection) showing ill-defined lytic and sclerotic lesions in tarsal bones with ankylosis at inter-tarsal and metatarsophalangeal joints.

Routine hematological and biochemical parameters were within normal limits, except for a reduced hemoglobin level of 9.8 g/dL. X-ray imaging of the foot revealed ill-defined lytic and sclerotic lesions in tarsal bones with ankylosis at inter-tarsal and metatarsophalangeal joints (Fig. 2, Fig. 3).

**Fig. 4:**
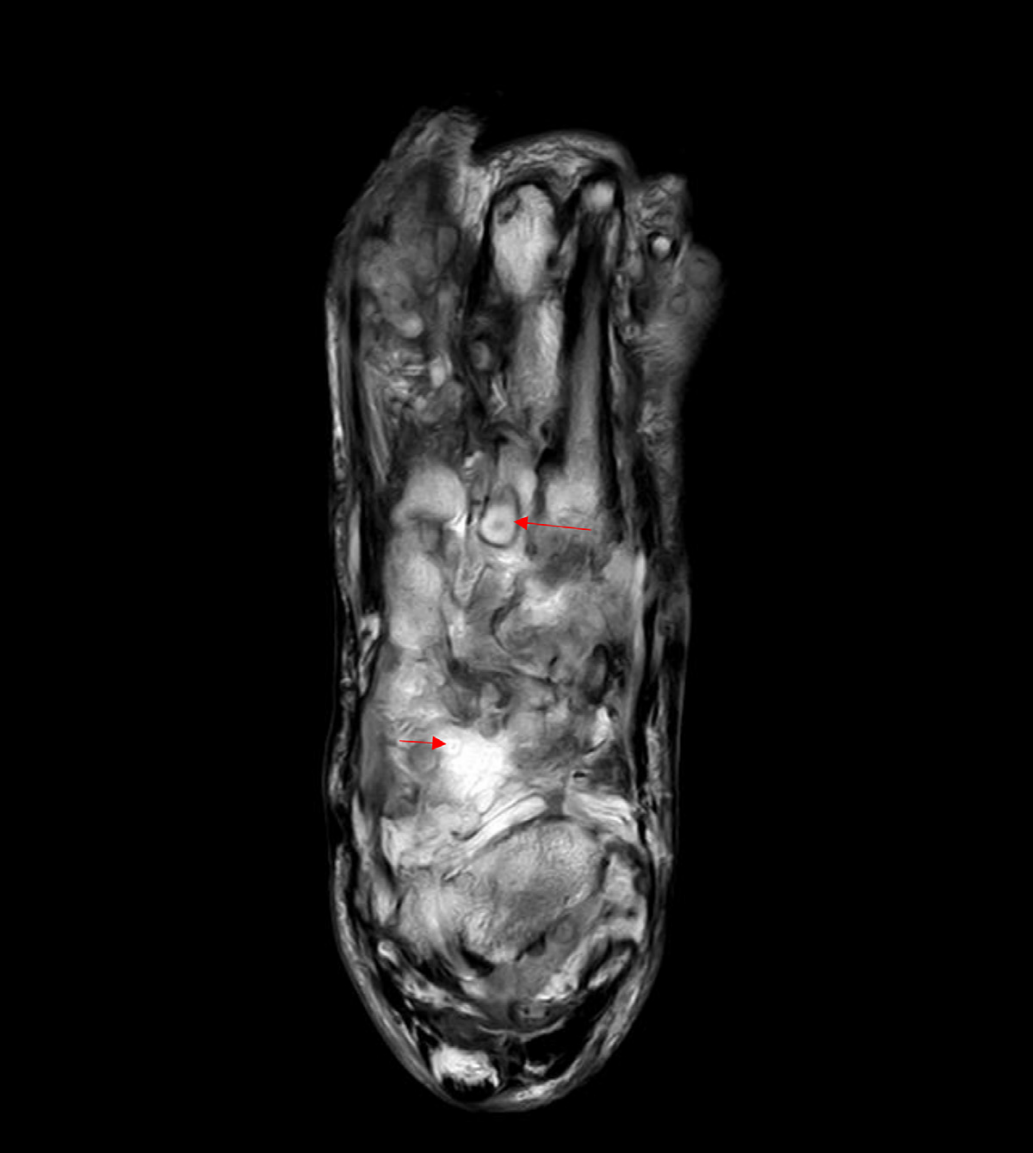
T2WI MRI image showing multiple enhancing lesions having central hyperintensity surrounded by a hypointense rim (dot in circle sign-red arrows), scattered along the intramuscular plane predominantly on the medial part of the dorsal aspect of the left foot.

**Fig. 5:**
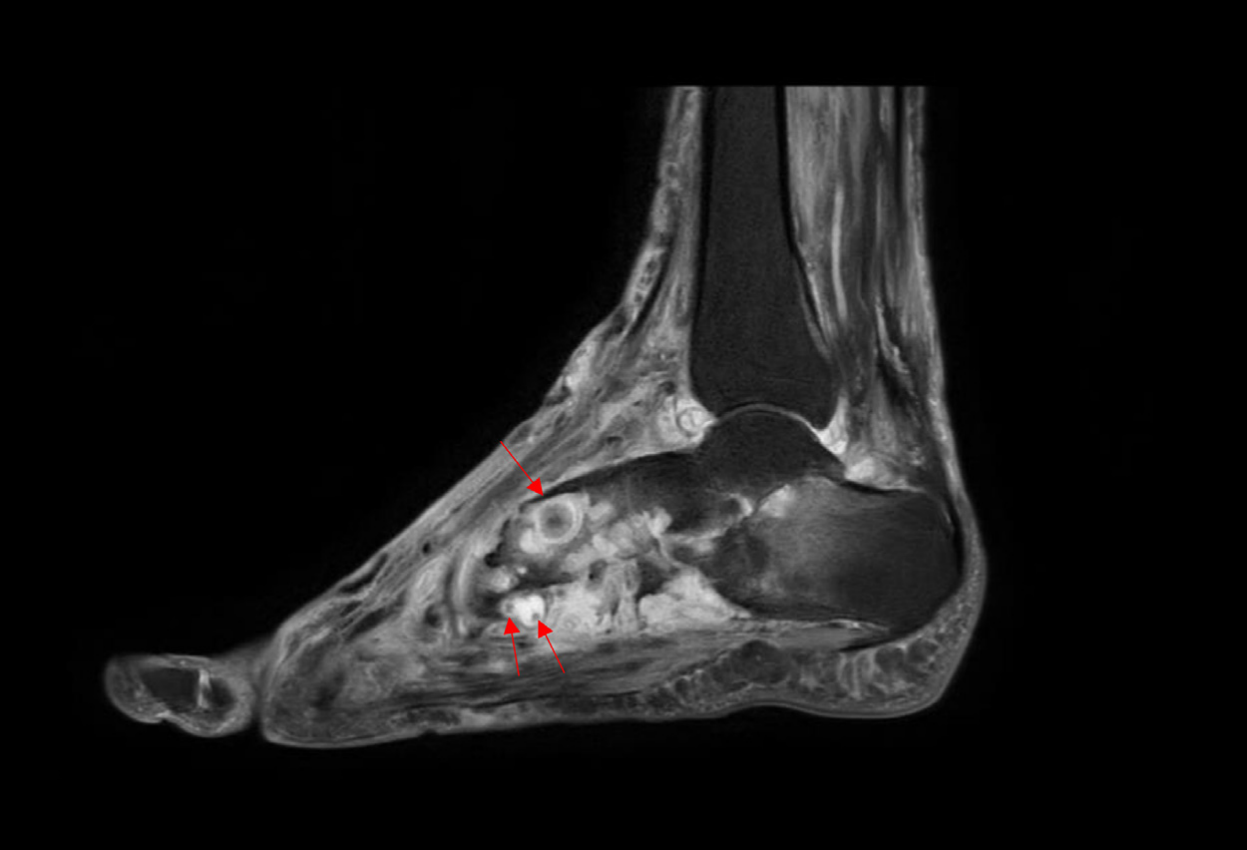
T2WI MRI image of left foot showing multiple enhancing lesions having central hyperintensity surrounded by a hypointense rim (dot in circle sign- red arrows), scattered along the intramuscular plane of foot. T2WI hyperintensity is seen in calcaneus s/o marrow edema due to infective etiology.

MRI showed multiple enhancing lesions having central hyperintensity surrounded by a hypointense rim on T2WI images (dot in circle sign), scattered along the intramuscular plane predominantly on the medial part of the dorsal aspect of the foot suggestive of fungal grains (Fig. 4, Fig. 5). Based on clinical presentation, examination, and imaging findings, we confirmed the diagnosis of eumycotic mycetoma.

## Discussion

A persistent, pseudotumorous infection of the skin and subcutaneous tissue that can also occasionally affect the bone is called a mycetoma. It is brought on by either bacteria (actinomycetoma) or fungi (eumycetoma). It is native to Mexico, India, and the tropics and subtropics of Africa. It has been reported that between 5.2% and 35% of mycetomas occur in India. It often manifests in individuals between the ages of 20 and 50, with a male-to-female ratio of 2.2:1 [1], [2], [3].

Deep penetrating traumatic injury to the skin and subcutaneous tissue inoculating the causative organism in the tissue is the first step in the pathogenesis of mycetoma. Following the pathogen's inoculation, an indolent subcutaneous infection ensues and advances very slowly over months to years. Young, healthy adults working in agriculture as well as people who frequently walk barefoot are affected by the condition [4,5]. The lower extremities, particularly the foot is the most often affected sites, with the hand coming in second. Areas including the arm, buttocks, shoulder, trunk, and neck are less frequently involved.

Reported cases have shown a range from a few months to sixty years between the time of the first infection and the final diagnosis [6].

Clinically, the disease follows an indolent but progressive course after initially presenting as a firm, painless nodule. Eventually, the lesions communicate via sinuses onto the skin surface or involve the adjacent bone to cause a form of osteomyelitis. Early diagnosis can be made based on the triad of features including painless swelling (tumefaction), draining sinuses, and purulent or seropurulent discharge with grains. However, the occurrence of all these findings is difficult. Biopsy or microbiological culture can provide the diagnosis, but this may not always be possible, especially if the organism is fastidious. Also, the techniques are invasive, time consuming, and technique sensitive and also due to unavailability of tissue diagnosis in the peripheral health setup, there is a delay in diagnosis and initiation of the treatment which can lead to significant destruction and deformity [6].

Imaging studies could aid in the management planning by determining the extension **of** the disease along and across tissue planes. The Mycetoma Research Centre and World Health Organization Collaborating Centre on Mycetoma have emphasized the importance of magnetic resonance imaging can be used to determine the extent of mycetoma lesions [7].

In a prospective study of MRI of 42 confirmed mycetoma patients, El Shamy et al. [8] proposed a grading system, The Mycetoma Skin, Muscle, Bone Grading System (MSMBS) to describe and grade disease severity on the basis of MRI findings. The mycetoma skin and subcutaneous plane findings, included fascial plane obliteration, abscess formation, and sinus tract formation with or without the presence of grains. Muscle plane findings included muscle edema, and formation of microabscesses or macroabscesses or both while bone plane findings, included bone marrow oedema, bone cavitation and bone destruction. Accordingly the mycetoma lesions were classified as mild (1-3), moderate (4-7) or severe (8-10). The study showed that the dot-in-circle sign, conglomerated foci with low signal intensity, and macro and micro-abscesses on a background of a hypointense matrix are all diagnostic of mycetoma.

The “dot-in-circle” sign on MRI has been postulated as the unique pathological features of mycetoma. The sign represents conglomeration of mycetoma causing micro-organisms known as “grains”, which are found within abscesses surrounded by abundant granulation tissue. It is described in MRI as conglomerates of small (2-5 mm) round hyperintense lesions, representing the granulation tissue, surrounded by low-signal-intensity rim, representing intervening fibrous septa. The central low-signal-intensity dot is the result of susceptibility effect caused by the presence of fungal grains [7], [8], [9], [10]. In our case, we noticed the aforementioned characteristic dot in circle sign.

## Conclusion

Our case report highlights the importance of MRI in the early diagnosis of mycetoma. The classical “dot-in-circle” sign aids to rule out other possible differential diagnosis.

## Patient consent

A written informed consent was obtained from the patient for publication of their case.
